# A comparison of the performance of ^68^Ga-Pentixafor PET/CT *versus* adrenal vein sampling for subtype diagnosis in primary aldosteronism

**DOI:** 10.3389/fendo.2024.1291775

**Published:** 2024-02-14

**Authors:** Xuan Yin, Kai Ai, Jianguang Luo, Wei Liu, Xiaowei Ma, Lianbo Zhou, Xin Xiang, Xin Su, Yunhua Wang, Yuan Li

**Affiliations:** ^1^ Department of Nuclear Medicine, The Second Xiangya Hospital, Central South University, Changsha, Hunan, China; ^2^ Department of Urology, The Second Xiangya Hospital, Central South University, Changsha, Hunan, China; ^3^ Department of Diagnostic and Interventional Radiology, The Second Xiangya Hospital, Central South University, Changsha, Hunan, China; ^4^ Department of Endocrinology, The Second Xiangya Hospital, Central South University, Changsha, Hunan, China

**Keywords:** primary aldosteronism, ^68^Ga-Pentixafor, PET/CT, CXCR4, endocrine hypertension

## Abstract

**Objective:**

To investigate the diagnostic efficiency and prognostic value of ^68^Ga-Pentixafor PET/CT in comparison with adrenal vein sampling (AVS) for functional lateralization in primary aldosteronism (PA). Histology and long-term clinical follow-up normally serve as the gold standard for such diagnosis.

**Methods:**

We prospectively recruited 26 patients diagnosed with PA. All patients underwent ^68^Ga-Pentixafor PET/CT and AVS. Postsurgical biochemical and clinical outcomes of patients with unilateral primary aldosteronism (UPA), as diagnosed by PET/CT or AVS, were assessed by applying standardized Primary Aldosteronism Surgical Outcome (PASO) criteria. Immunohistochemistry (IHC) was performed to detect the expression of aldosterone synthase (CYP11B2) and CXCR4.

**Results:**

On total, 19 patients were diagnosed with UPA; of these, 13 patients were lateralized by both PET/CT and AVS, four patients were lateralized by PET-only, and two by AVS-only. Seven subjects with no lateralization on AVS and PET received medical therapy. All patients achieved complete biochemical success except one with nodular hyperplasia lateralized by AVS alone. The consistency between PET/CT and AVS outcomes was 77% (20/26). Moreover, CYP11B2-positive nodules were all CXCR4-positive and showed positive findings on PET. Patients who achieved complete biochemical and clinical success had a higher uptake on PET as well as stronger expression levels of CXCR4 and CYP11B2.

**Conclusion:**

Our analysis showed that ^68^Ga-Pentixafor PET/CT could enable non-invasive diagnosis in most patients with PA and identify additional cases of unilateral and surgically curable PA which could not be classified by AVS. ^68^Ga-Pentixafor PET/CT should be considered as a first-line test for the future classification of PA.

## Introduction

Primary aldosteronism (PA), the most frequent cause of secondary arterial hypertension, exerts a significant impact on healthcare (1). Compared with equal grade essential hypertension, PA leads to a significantly higher extent of cardiovascular morbidity, with an increased risk of strokes, atrial fibrillation, and heart failure (2, 3). However, this condition can be cured with appropriate treatment. Adrenalectomy is recommended for patients with unilateral primary aldosteronism (UPA); this is associated with a higher rate of cure (4) while bilateral primary aldosteronism (BPA) is usually treated with mineralocorticoid-receptor antagonists (MRAs) (5). Therefore, it is essential to classify PA accurately for individually optimized therapy.

Nevertheless, only a minority of patients receive appropriate management because the diagnostic process remains challenging (6). At present, adrenal venous sampling (AVS) is widely accepted as the reference standard for functional assessment of adrenal masses (2, 7). However, not only is it an invasive and technically challenging approach, AVS also carries a risk of serious complications, thus limiting its application in clinical practice on PA subtyping (5, 8, 9).

Functional imaging in nuclear medicine, known as molecular imaging technology, has been proven to have significant potential. 11C-metomidate, as a positron emission tomographic (PET) imaging tracer, has been used for the diagnosis of primary aldosteronism in several small studies (10–17). Nevertheless, the low selectivity for CYP11B2 (aldosterone synthase) as well as CYP11B1 (11β-hydroxylase) and the short radio half-life of 11C presents challenges in this procedure (18).

The C-X-C chemokine receptor type 4 (CXCR4) is a transmembrane G protein-coupled receptor; the expression of this protein has been reported to be upregulated in aldosterone-producing tissue but is almost negligible in non-functional adenoma(NFA) (19–21). The PET tracer gallium-68 Pentixafor (^68^Ga-Pentixafor) is a specific ligand for CXCR (22–24). Recent research demonstrated that ^68^Ga-Pentixafor PET/CT possesses unprecedented accuracy for the detection of aldosterone-producing adenoma (APA), mainly based on histopathology (25–30). However, there are only few reports assessing the concordance of ^68^Ga-Pentixafor PET/CT and AVS, the current gold standard. As complete biochemical success could define the correct diagnosis and appropriate treatment (31–33), in the present study, we compared PET with AVS by using biochemical outcomes as a primary quality measure of diagnosis to evaluate its efficacy for identifying functional adrenal adenomas. This study aimed to explore the potential of ^68^Ga-Pentixafor PET as a noninvasive alternative to AVS to help guide clinical management decisions.

## Methods

### Study design and participants

The study protocol was approved by Ethics Committee of National Medical Research Center, Second Xiangya Hospital, Central South University. All patients provided written informed consent prior to ^68^Ga-Pentixafor PET/CT.

**Figure 1:**
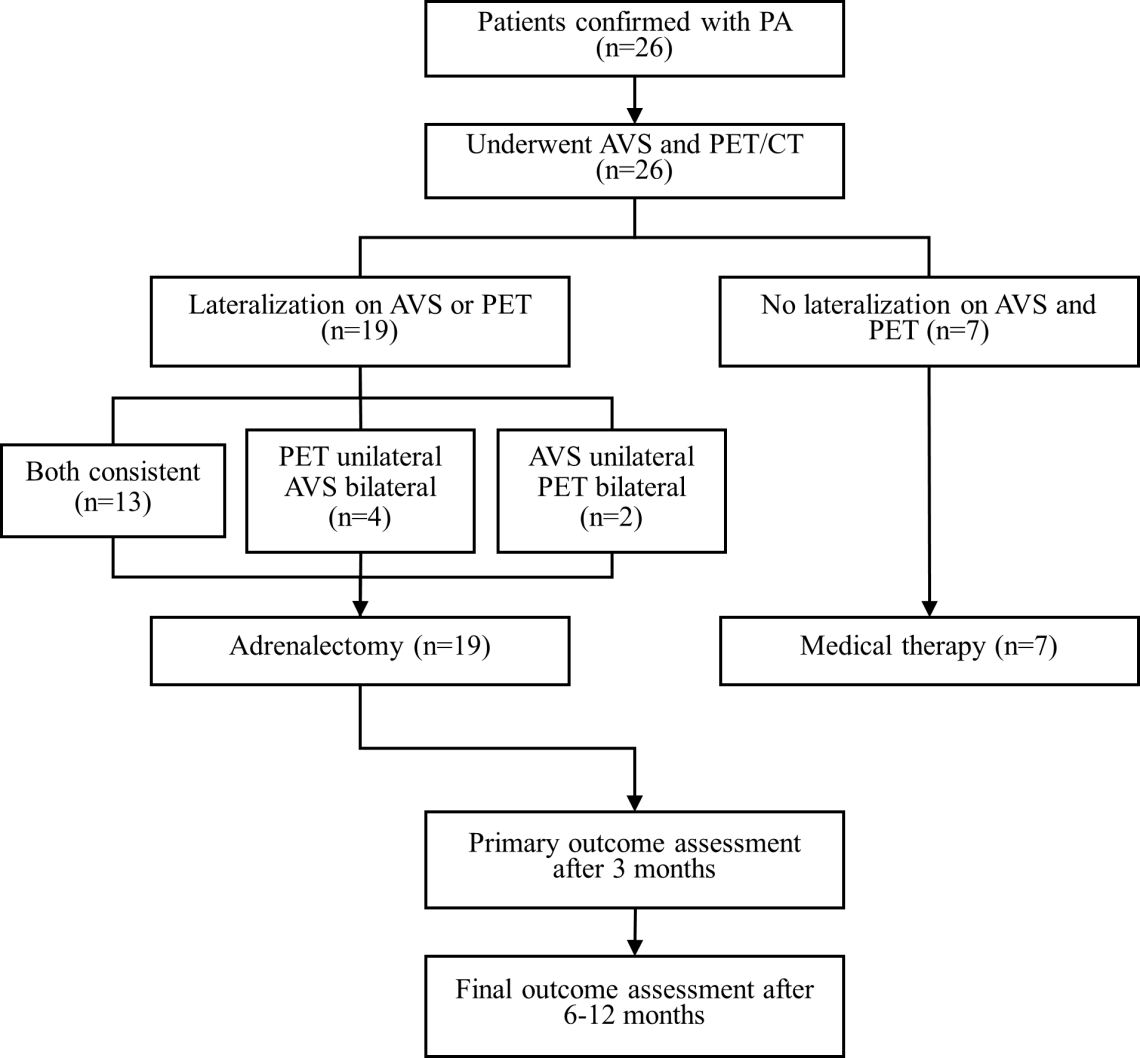
Results of AVS, ^68^Ga-Pentixafor PET/CT and treatment of all patients. AVS, adrenal vein sampling; CT, computed tomography; PET, ^68^Ga-Pentixafor PET/CT.

This was a prospective clinical trial that used both ^68^Ga-Pentixafor PET/CT and AVS to subtype PA. We prospectively recruited patients with a clinical diagnosis of PA at The Second Xiangya Hospital from the 1^st^ of July 2022 to the 1^st^ of December 2022. The patients were referred to us by a certified panel of specialists in clinical endocrinology. Patients were eligible if they had confirmed PA (according to guidelines published by the Endocrine Society, details are provided in the **Supplementary Materials**) and were scheduled for adrenalectomy (33–36). The exclusion criteria were as follows: 1) a suspicion of adrenocortical carcinoma; 2) familial PA due to germline mutations, and 3) contraindications to isotope scanning, for example pregnancy or claustrophobia. Finally, 26 patients were included in our analysis (**Figure 1**).

### Adrenal vein sampling

Patients with confirmed PA underwent adrenal CT scanning and AVS. Prior to the AVS procedure, medications that may influence the levels of renin and aldosterone (such as potassium sparing diuretics, aldosterone receptor antagonists, and angiotensin receptor-blockers) were withdrawn at least for 2 to 6 weeks. We corrected hypokalemia as early as possible, if present, to reach a serum potassium level that was ≥3.5 mmol/L. Detailed methods and criteria are provided in the **Supplementary Materials**.

### ^68^Ga-Pentixafor synthesis

^68^Ga-Pentixafor was synthesized in a sterile environment following reported labelling approach (30, 37). Details are provided in the **Supplementary Materials.**

### PET/CT imaging and analysis

^68^Ga-Pentixafor PET/CT scans were acquired by a dedicated PET/CT scanner (Siemens Biograph mCT 64; Siemens Medical Solutions, Erlangen, Germany). Prior to the acquisition of ^68^Ga-Pentixafor PET/CT images, the patients received a normal diet with no special preparation. After 25 min of the intravenous administration of ^68^Ga-Pentixafor (mean 88 ± 15 MBq), static images were collected from the head to the mid-thigh for 10 min. Corresponding CT scans for attenuation correction were acquired over the adrenal glands using a low-dose protocol using specific parameters (35 mAs, 120 keV, a 512 × 512 matrix, a 5-mm slice thickness, an increment of 5 mm/s, a rotation time of 0.5 s, and a pitch index of 0.8). Dynamic images were reconstructed using the following scheme: 1 × 40 s, 10 × 5 s, 3 × 10 s, 2 × 15 s,5 × 30 s, 5 × 120 s, 5 × 300 s and 2 × 600 s. Fused PET and low-dose CT images were obtained to evaluate the uptake of ^68^Ga-Pentixafor.

All PET scans were independently analyzed by two nuclear medicine physicians experienced in ^68^Ga-Pentixafor PET interpretation. These physicians were blinded to clinical data and AVS results. Disagreements were decided by mutual consensus. A positive adrenal lesion detection by PET/CT was defined by visual analysis as exhibiting a higher uptake than the ipsilateral or contralateral normal adrenal glands. A negative detection was considered if there was an equal or reduced uptake of ^68^Ga-Pentixafor when compared with the contralateral adrenal glands. Normal adrenal glands were defined as those with no morphological changes in the contralateral to unilateral lesions of PA patients who achieved complete biochemical success after adrenalectomy.

Quantitative analyses were performed with PMOD 4.3 software (Zurich, Germany: PMOD Technologies). We also calculated the maximum standardized uptake values (SUVmax) of the adrenal lesions, specific uptake value ratios such as the lesional SUVmax to the normal liver SUVmean (LLR), and the lesional SUVmax to the contralateral adrenal tissue SUVmean (LCR).

### Management of patient therapy and outcomes

Patients underwent AVS and then ^68^Ga-Pentixafor PET/CT; the mean time interval between these tests were 3 ± 1 days (range, 2–4 days). The management of patients was co-determined at a multidisciplinary meeting of endocrinologists, radiologists and urologists, based on clinical and imaging presentations. If one of the AVS or PET/CT images displayed lateralization, the patient would undergo unilateral adrenalectomy. If neither of the images were lateralized, medication was administered.

The outcome was first evaluated approximately three months after adrenalectomy in accordance with the Primary Aldosteronism Surgical Outcome (PASO) consensus (**Supplementary Table 1**) (31). For those treated by medical therapy, we collected medicine and blood pressure data for comparison. At least six months post-treatment, patients were reassessed for biochemical and clinical outcomes. The primary outcome was the lateralization accuracy of PET in comparison with AVS by considering the biochemical cure rate post-surgery as the reference. Secondary outcome was the accuracy of each diagnostic test compared to final histology and clinical follow-up.

### Immunohistochemistry

Immunohistochemical analyses were performed using paraffin-embedded specimens from 19 subjects who underwent unilateral adrenal excision. CXCR4 and CYP11B2 antibodies were used as primary antibodies. Immunohistochemical staining was performed on adrenal sections with an automatic immunostaining system. Detailed semi-quantitative analysis are provided in the **Supplementary Materials**. Classical (unilateral aldosterone-producing adenoma, APA) and non-classical (multiple aldosterone-producing nodules/micronodules, MAPN/MAPM or aldosterone-producing diffuse hyperplasia, APDH) PA were diagnosed according to the Histology of Primary Aldosteronism (HISTALDO) consensus (37, 38).

### Statistical analysis

This study was designed to have a power of 80% to detect a sensitivity of 0.8 when comparing to a non-significant diagnosis (a sensitivity of 0.5) using a two-sided test at a significance level of 0.05. First, normality was assessed using the S-W test. Nonnormally distributed data were expressed as median (interquartile range, IQR) and compared using the Mann-Whitney U-test, while normally distributed data were reported as mean ± SD and compared using unpaired t test. The Chi-squared test was used for categorical variables. The correlation between the uptake and uptake ratio of ^68^Ga-Pentixafor in adrenal lesions as well as other characteristics of patients were assessed using Pearson’s or Spearman’s correlation coefficient. Receiver-operating characteristic (ROC) curves were also constructed to determine the threshold for semi-quantitative parameters and the diagnostic accuracy of ^68^Ga-Pentixafor PET/CT for the diagnosis of UPA, with complete biochemical success as the gold standard. Statistical significance was defined as P < 0.05.

## Results

### Baseline characteristics

In total, 26 patients (eight females and 18 males) were included in this investigation. The mean age of the patients was 50.3 ± 2.0 years and the median duration of hypertension was 8.5 years. All patients with PA (100%, 26/26) suffered from hypertension; four of these patients (15%, 4/26) had refractory hypertension. Twenty-four patients with PA (92%, 24/26) had hypokalemia. **Table 1** shows detailed characteristics. Individual data of all 26 patients recruited into study is provided in **Supplementary Table 2**.

**Table 1:** Characteristics of patients recruited in study.

Characteristics	Total (n=26)	UPA (n=19)		BPA (n=7)	
		Baseline	Post-treatment	Baseline	Post-treatment
Age (years)	50.3±2.0	49.6±2.6	–	52.0±3.0	–
Gender, Female/Male	8/18	7/12	–	1/6	–
BMI (kg/m2)	25.1±1.0	25.3±0.9	–	26.2±0.8	–
Duration of hypertension (years)	8(3-12)	10(3-15)	–	7(6-8)	–
Systolic BP (mmHg)	183±8	181±6	127±4 ****	172±7	131±2 ***
Diastolic BP (mmHg)	110±5	108±4	80±2 ****	98±3	82±2 **
Duration of hypokalemia (years)	0.5(0.02-2)	0.5(0.02-2)	–	0.5(0.08-1)	–
Serum potassium (mmol/l)	2.7±0.1	2.6±0.2	4.2±0.1 ****	2.9±0.2	4.1±0.1 ***
PAC (ng/dl)	33.3(21.9-47.8)	33.5(21.6-57.0)	3.7(3.1-8.2) ****	34.2±4.9	8.3±1.7 **
PRA (ng/ml/h)	0.1(0.07-0.3)	0.2±0.04	0.4(0.2-1.8) **	0.1 (0.04-0.2)	0.6(0.4-0.7)
ARR ([ng/dl]/[ng/ml/h])	176.3(112.3-417.7)	167.4(103.1-415.0)	10(4.6-18.8) *	468.9±182.4	12.5±2.1 *
Long diameter on CT (cm)	1.2(0.7-2.5)	1.4±0.1	–	1.0±0.1	–
AVS-LI	11.6±3.4	7.5(3.8-38.9)	–	1.3±0.1	–

****P <0.0001; ***P <0.001; **P <0.01; *P <0.05. (Asterisks indicate significant differences between baseline vs post treatment).

UPA, unilateral primary aldosteronism; BPA, bilateral primary aldosteronism; BMI, body mass index; BP, blood pressure; PAC, plasma aldosterone concentration; PRA, plasma renin activity; ARR, aldosterone-renin ratio; AVS-LI, lateralization index based on AVS.

### Clinical management

All 26 patients underwent ^68^Ga-Pentixafor PET/CT and AVS. Nineteen patients were diagnosed with UPA and underwent adrenalectomy. Of these, 15 cases of APA and four cases of MAPN/MAPM were definitively diagnosed by histology and immunohistochemistry. The remaining seven subjects with no lateralization on AVS and PET were considered to have BPA and subsequently received medical therapy.

### Comparisons between AVS and ^68^Ga-Pentixafor PET/CT

Using the PASO consensus, of the 19 patients who underwent adrenalectomy, 18 patients achieved complete biochemical success and only one patient achieved partial biochemical success. This patient had a diagnosis of right-sided dominant aldosterone secretion by AVS but not PET and was confirmed to have nodular hyperplasia by pathological examination. All subjects showed an improvement of blood pressure; 15 had complete clinical success and four showed partial success (two were diagnosed by PET and AVS, one by PET-only, and one by AVS-only). None of the patients showed an absent biochemical or clinical success.

**Figure 2:**
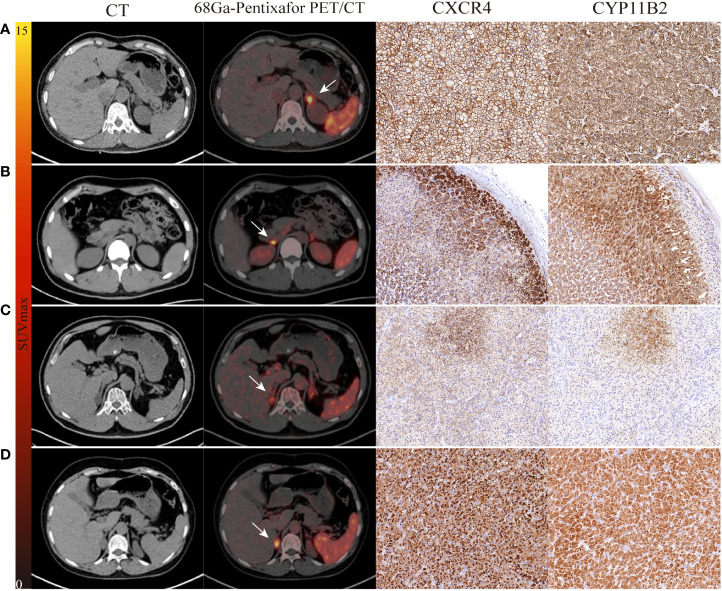
The performance of ^68^Ga-Pentixafor PET/CT imaging in PA patients. **(A, B)** show strong and moderate expression separately, as determined by immunohistochemistry with CXCR4 and CYP11B2; positive findings were detected on both AVS and PET/CT scanning (LI, SUVmax; 21.27, 13.01 vs. 8.10, 5.54). **(C)** A 54-year-old male with bilateral adrenal gland lesions on CT. AVS lateralized the right (LI of 6.73) while PET/CT showed comparable uptake on both sides (SUVmax of R-8.81 and L-7.86). Postoperative pathological examination identified MAPM with weak expression of CXCR4 and CYP11B2. Partial biochemical and clinical success were observed during follow-up. **(D)** A 60-year-old female with concurrent hypercortisolism. AVS was indefinite (LI of L-1.07 and R-0.93) while PET/CT showed positive finding (SUVmax of 14.42); there was positive expression of CXCR4 and CYP11B2. White arrows indicate the tumor lesion. Magnification ×20 for immunohistochemical staining.

**Figure 3:**
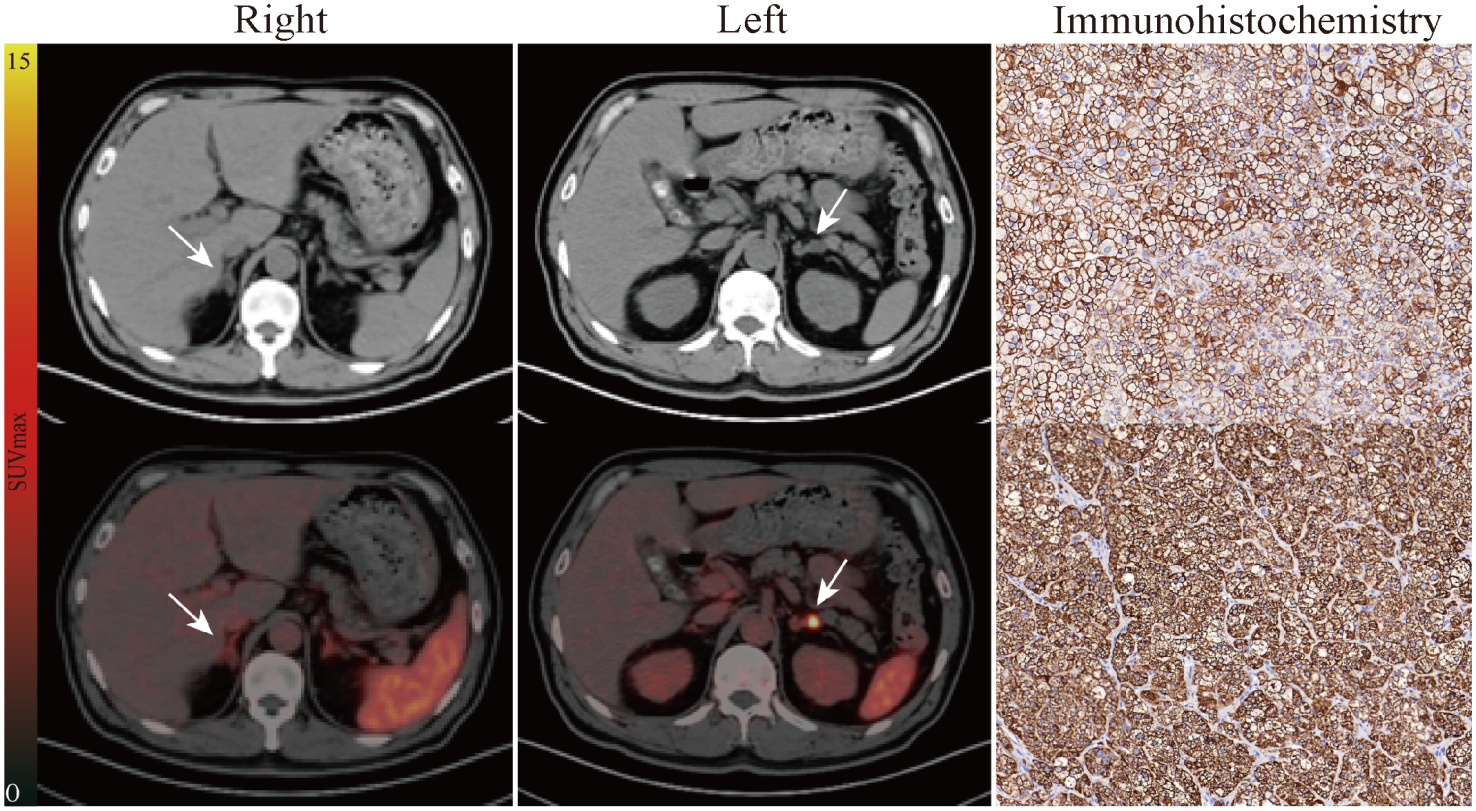
Representative pathological and imaging findings from a 54-year-old male with bilateral adrenal nodules. CT (up) and fusion image (down) in the "Right" column showed slight radioactivity uptake in the right adrenal lesion (1.5 cm × 1.0 cm, SUVmax of 2.86). Left lateralization was identified by ^68^Ga-Pentixafor PET/CT as is shown in the "Left" column (1.5 cm × 1.1 cm, SUVmax of 5.13, LCR of 1.79, LLR of 3.25). AVS showed the same judgment (LI of 3.80). The patient subsequently underwent left adrenalectomy. Immunohistochemistry for CXCR4 (up) and CYP11B2 (down) showing high levels of expression. Follow-up confirmed complete biochemical success. White arrows indicate the tumor lesion. Magnification ×20 for immunohistochemical staining. LCR, ratio of lesional SUVmax to contralateral adrenal SUVmean; LLR, ratio of lesional SUVmax to normal liver SUVmean.

The consistency between PET and AVS findings was 77% (20/26). Both ^68^Ga-Pentixafor PET/CT and AVS examinations consistently and accurately identified 13 cases of UPA and seven cases of BPA. None of the patients showed AVS-determined lateralization that was contralateral to the lesion on PET/CT. Of the 19 UPA patients, 17 cases were lateralized correctly by ^68^Ga-Pentixafor PET while 15 were identified by AVS. Four patients showed no dominant secretion on initial AVS but PET imaging showed unilateral lateralization (three on the left and one on the right), including one patient with concurrent hypercortisolism; all patients achieved biochemical success following unilateral adrenal resection. In addition, two patients had only AVS-determined lateralization. One of the patients with bilateral nodules on CT showed comparable ^68^Ga-Pentixafor uptake on both sides while AVS only identified uptake on the right (**Figure 2**). Of the 10 patients presenting with bilateral adrenal gland lesions, ^68^Ga-Pentixafor PET/CT successfully identified functional lateralization in nine patients, whereas AVS lateralized eight subjects (**Figure 3**). Characteristics of six patients with discordant lateralization in PET/CT and AVS was shown as **Supplementary Table 3**.

**Figure 4:**
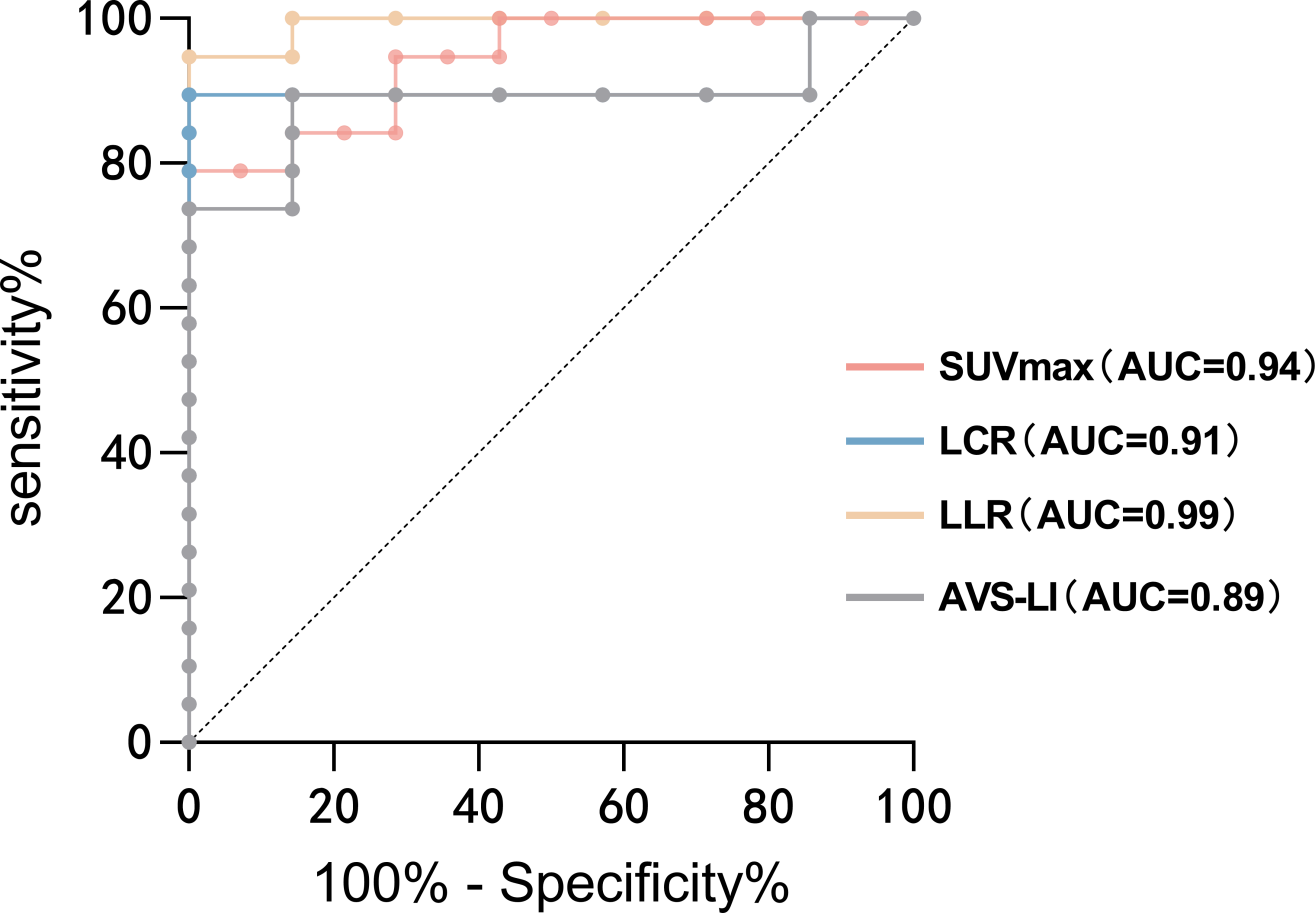
ROC analysis for identifying the dominant side of PA. The AUCs of ROC curves for SUVmax, LCR, LLR and AVS-LI were 0.94 (95% CI, 0.87–1.00), 0.91 (95% CI, 0.79–1.00), 0.99 (95% CI, 0.97–1.00) and 0.89 (95% CI, 0.75–1.00), respectively. To diagnose UPA, the LLR had a higher AUC than other uptake values of PET/CT and AVS-LI. AUC, the area under the ROC curve; AVS-LI, lateralization index based on AVS.

An optimal cutoff SUVmax value of 5.71 was calculated by ROC analysis yielding a sensitivity of 78.95%, a specificity of 100%. The area under the ROC curve (AUC) was 0.94 (95% CI, 0.87–1.00). A cutoff value for LCR of 1.39 yielded a sensitivity of 89.47% and a specificity of 100%, whereas the cutoff value for LLR at 3.05 yielded a sensitivity of 94.74% and a specificity of 100%. The AUC for LCR and LLR were 0.91 (95% CI, 0.79–1.00) and 0.99 (95% CI, 0.97–1.00), respectively. Moreover, the AUC for AVS-LI was 0.89 (95% CI, 0.75–1.00). To diagnose UPA, the LLR had a higher AUC than other uptake values of PET/CT and AVS-LI (**Figure 4**).

### Correlation of ^68^Ga-Pentixafor PET/CT with clinical management and outcomes

Patients in the surgery group had a high ^68^Ga-Pentixafor SUVmax on the dominant side compared with those who received medications (13.2 ± 1.9 vs 3.9 ± 0.5, p < 0.01). Similarly, significantly higher LCR and LLR values were evident in the surgery group when compared with the medication group (3.2 ± 0.5 vs 1.3 ± 0.1; 7.7 ± 1.0 vs 2.4 ± 0.3, respectively, p < 0.05).

**Figure 5:**
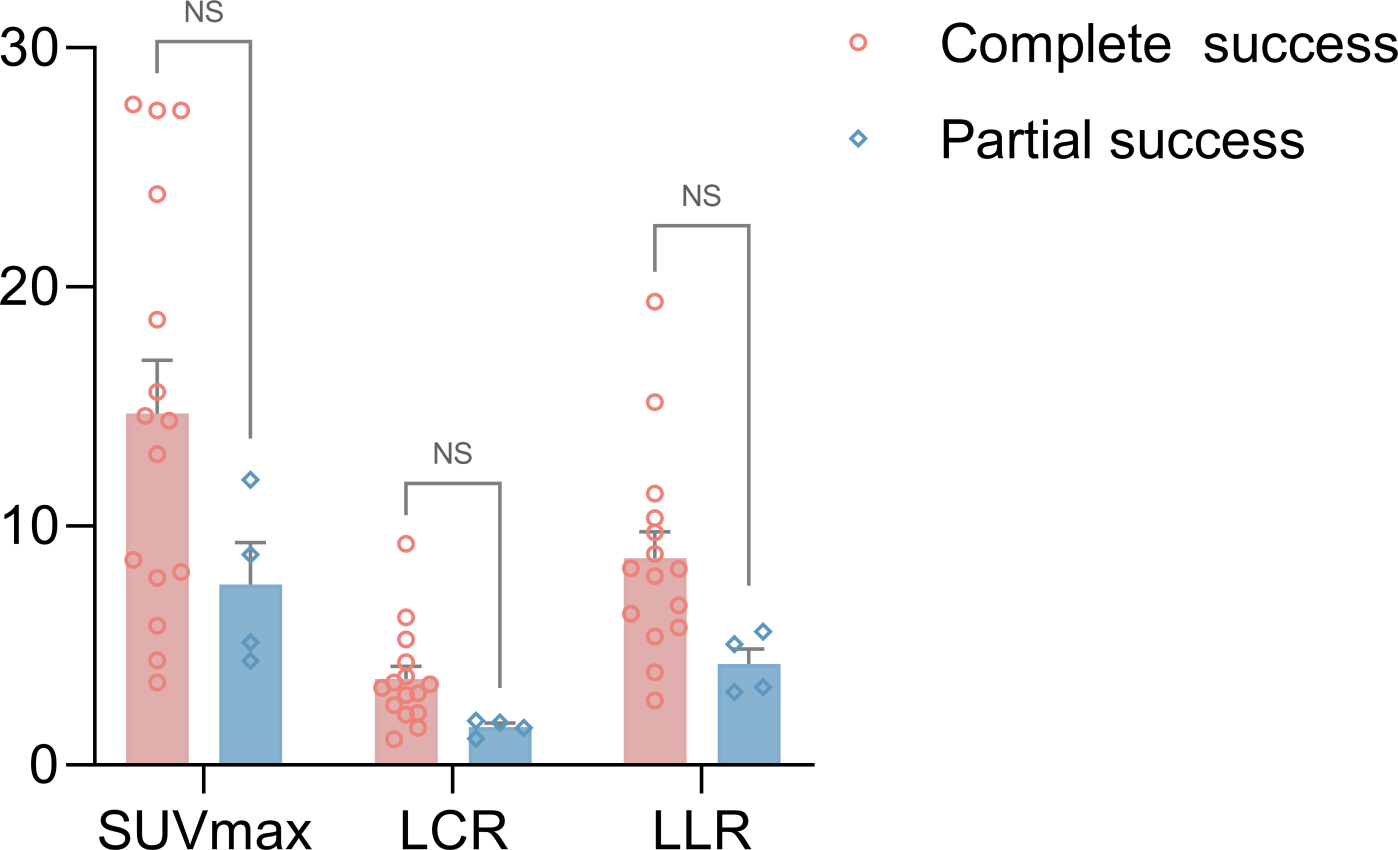
A comparison of ^68^Ga-Pentixafor SUVmax, LCR and LLR values between the complete biochemical and clinical success group and the partial success group for surgery patients (SUVmax of 14.7 ± 2.2 vs. 7.5 ± 1.7; LCR of 3.6 ± 0.5 vs. 1.6 ± 0.2; LLR of 8.6 ± 1.1 vs. 4.2 ± 0.6, respectively, p > 0.05). ns, no significance.

Among the surgery group, patients who achieved both complete biochemical and clinical success had higher uptake values for resected adrenal lesions than those who achieved partial success (**Figure 5**). The SUVmax, LCR, and LLR of ^68^Ga-Pentixafor were 14.7 ± 2.2 versus 7.5 ± 1.7 (p = 0.12), 3.6 ± 0.5 versus 1.6 ± 0.2 (p = 0.07), and 8.6 ± 1.1 versus 4.2 ± 0.6 (p = 0.06), respectively. However, the difference was not statistically significant.

### Pathological and immunohistochemical analysis

Immunohistochemical analysis was performed for CXCR4 and CYP11B2 in sections of postoperative adrenal tissue from 19 patients with UPA. Immunohistochemical tests for CYP11B2 were used to identify functional nodules. All lesions (19/19, 100%) had CYP11B2-positive nodules. Based on this data, 15 subjects were classified as having classical unilateral primary aldosteronism–APA, of whom 11 were lateralized by both PET/CT and AVS; three patients had PET-only lateralization and one had AVS-only lateralization. All patients showed complete biochemical success post-surgery. Furthermore, MAPN/MAPM were discovered in the remaining four lesions. Of these, two cases were consistently lateralized by PET/CT and AVS and one was lateralized by PET and achieved complete biochemical success. One patient with bilateral multiple adrenal nodules was lateralized by AVS alone and demonstrated bilateral comparable radioactive uptake on PET/CT; this patient achieved partial biochemical success after surgery. Based on the adrenal glands, the highest uptake value for ^68^Ga-Pentixafor uptake was detected in typical APA lesions (**Supplementary Figure 1**).

**Figure 6:**
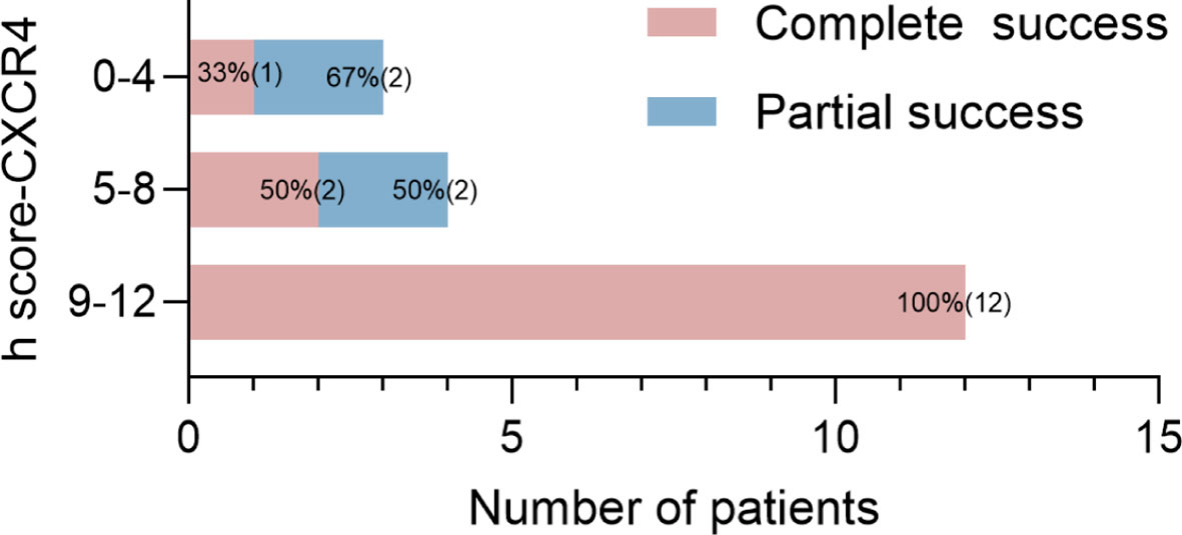
Proportions and absolute numbers (in parentheses) of patients with different prognoses for the three groups by h score (0–4, 5–8, 9–12).

Moreover, CYP11B2-positive nodules were all CXCR4-positive and showed positive findings on ^68^Ga-Pentixafor PET/CT. The h score of CXCR4 and CYP11B2 showed a significant relationship with the SUVmax of ^68^Ga-Pentixafor (r = 0.56, 0.54, respectively, p < 0.05). Furthermore, patients with complete biochemical and clinical success had a higher h score for CYP11B2 by immunohistochemistry than those with partial success (p < 0.001). The higher h score for the CXCR4 group indicated a significantly higher rate of complete success in patients than the low score group (**Figure 6**).

### Correlation between ^68^Ga-Pentixafor PET/CT and clinical characteristics

The results of the correlation analyses for SUVmax for ^68^Ga-Pentixafor uptake with lesions and patient clinical features among the 26 patients are shown in **Table 2** and **Supplementary Figure 2**. The long diameter of nodules exhibited a moderate positive association with SUVmax (Spearman ρ = 0.47, p < 0.05). A moderate correlation was detected between PAC and SUVmax (Spearman ρ = 0.42; p < 0.05). Furthermore, LI based on AVS was significant and positively correlated with the SUVmax (Spearman ρ = 0.74; p < 0.01) of dominant adrenal glands in patients diagnosed with UPA. The relationships of other ^68^Ga-Pentixafor uptake values with clinical features are shown in **Supplementary Table 4**.

**Table 2:** Correlation coefficients between SUVmax of PA patients and clinical features.

Clinical features	Correlation coefficients	P value
Age	-0.31	0.13
BMI	-0.08	0.71
Systolic pressure	0.12	0.57
Diastolic pressure	0.33	0.01
The long diameter of nodule	0.47*	0.02
AVS-LI^#^	0.74**	0.001
Serum potassium	-0.03	0.89
PAC	0.42*	0.04
ARR	-0.09	0.66

**P <0.01; *P <0.05. #: among patients who were diagnosed with UPA.

## Discussion

In this prospective clinical study, non-invasive ^68^Ga-Pentixafor PET/CT demonstrated comparable functional detection capabilities for subtyping PA when compared with AVS using biochemical and clinical follow-up as the gold standard. Notably, both methods independently detected PA patients who could be cured by unilateral adrenal resection, while the other was not successfully lateralized. The sensitivity and accuracy of ^68^Ga-Pentixafor PET for the functional lateralization of PA patients were 89% (17/19) and 92% (24/26) respectively, while those of AVS were 79% (15/19) and 85% (22/26), respectively.

Our results showed that the optimum SUVmax cut-off for the identification of functional nodules based on ^68^Ga-Pentixafor was 5.71; at this cut-off value, the sensitivity was 78.95% while the specificity was 100%. The AUC was 0.94 (95% CI, 0.87–1.00). However, the absolute values of SUVmax among functional lesions varied widely from patient to patient; this may be attributed to different CXCR4 expression levels between individuals. Referring to the dominant side diagnostic principle of AVS, the uptake value ratios should play a greater role in classification. As shown by our data, the LCR and LLR performed better than SUVmax. Notably, when the threshold of LLR was 3.05, the sensitivity and specificity were 94.74% and 100%, respectively; the AUC was 0.99. This finding is in agreement with a previous prospective study involving 33 PA and 3 NFA patients which demonstrated the superior detectability of LLR than other uptake values (29). The consistency between this quantitative criteria and our initial “visual assessment” was 96% (25/26). Thus, LLR might be considered as the best index of ^68^Ga-Pentixafor PET/CT for the identification of functional lesions.

During follow-up, 18 patients who underwent adrenalectomy achieved complete biochemical cure; of these, 17 patients (94%, 17/18) were identified by PET/CT and 14 patients (78%, 14/18) were identified by AVS. The consistency of these two methods was 77% (20/26). We found that four UPA patients presented with false-negative results on AVS while true-positive lesions were identified by PET/CT scanning. All of these achieved biochemical cure after excision of the dominant adrenal gland on PET, including one patient with concurrent hypercortisolism and another who had adrenal poly-nodular hyperplasia post-surgery. Furthermore, three of these patients showed bilateral adrenal gland lesions on CT. These results showed that ^68^Ga-Pentixafor could be used to diagnose UPA cases where AVS results are not definite or non-identifiable. Nevertheless, two UPA patients with a true-positive result on AVS showed a comparable uptake value on both sides in PET/CT. Postoperative pathology from one of these patients confirmed nodular hyperplasia; subsequent immunohistochemical staining of CXCR4 and CYP11B2 indicated low levels of MAPM expression. Partial biochemical success was observed. The other patient had an adenoma that was < 8 mm in size; the LI was close to the threshold.

There were six patients with discordant lateralization in PET/CT and AVS. Though few in number, we found that 50% (3/6) patients presented with bilateral adrenal gland lesions (nodules or hyperplasia) on CT. Of the remaining patients with unilateral adrenal disease, nobody has classical single nodule except for one with concurrent hypercortisolism. Compared to consistent cases, the proportion of multiple nodules and hyperplasia is significantly higher. We speculate the reason for discordance cases may be that these patients have asymmetrical bilateral disease, and the two methods detect different levels of lesions functionality in different way, leading to inconsistent lateralization results. Besides, for adrenal glands that appear “normal” on CT, functional changes may precede morphological changes. The smallest nodule size that can be detected is 0.7cm in our study. And there is a moderate positive association between SUVmax of PET/CT and the long diameter of adrenal nodules (Spearman ρ = 0.47, p < 0.05), suggesting that the sensitivity of this method may be more ideal in patients with bigger lesions.

In our study, all four hyperplastic adrenal lesions with positive uptake on ^68^Ga-Pentixafor PET/CT showed increased expression levels of CXCR4 and CYP11B2. Even if the h score was lower than classical APA, it can be inferred that the hyperplastic adrenal tissue in patients with PA may also be functional and could be recognized by ^68^Ga-Pentixafor PET/CT. Moreover, our data are supported by the latest World Health Organization (WHO) Classification of Adrenal Cortical Tumors, which recommends using CYP11B2 immunohistochemistry to identify the functional sites of aldosterone production rather than simply distinguishing functional adenomas from non-functional hyperplasia (34).

In addition, all 19 UPA cases undergoing immunohistochemical staining were positive for CYP11B2 and all CYP11B2-positive lesions were also CXCR4-positive. In semi-quantitative analysis, SUVmax was correlated with the h score for both CXCR4 and CYP11B2 (r = 0.56, 0.54, respectively, p < 0.05). Furthermore, we found patients who achieved both complete biochemical and clinical success had higher h scores for CXCR4 and CYP11B2, as determined by immunohistochemistry of resected adrenals, when compared with those who achieved partial success (p < 0.001). CYP11B2 is involved in the terminal steps of aldosterone biosynthesis and, allows the localization of aldosterone synthesis. However, the precise relationship between radioactive uptake and the secretion of aldosterone remains unclear and further studies are now required.

An important consideration is whether non-invasive functional imaging ^68^Ga-Pentixafor PET/CT, as a first differential diagnostic step, might enable non-invasive diagnosis in most patients with PA. According to our data, 17 patients (89%, 17/19) were successfully diagnosed as UPA by PET, thus avoiding invasive AVS. Only two patients with UPA missed the opportunity for surgery. When considering patients without obvious PET lateralization, it was evident that these patients could choose to receive medication or proceed with subsequent AVS as a second-line examination. Unlike the high technical requirements of AVS, ^68^Ga-Pentixafor PET/CT is readily available at most centers where PET scans can be performed. And none of patients reported adverse events associated with ^68^Ga-Pentixafor. Furthermore, as an outpatient procedure, PET/CT can save considerable amounts of time and economic costs than AVS for patients. In the healthcare centers of China, the average cost of AVS was about ¥12000, while that of PET/CT is ¥5500. According to the published literature, the average price of PET/CT in Europe is also about half the average price of AVS (≈€1200). The application of new technology can save patients about half of the previous cost while eliminating the pain of surgery. In our patient satisfaction survey, all subjects expressed a positive attitude towards the replacement of invasive tests with non-invasive tests in the future. Based on these findings, we can conclude that ^68^Ga-Pentixafor PET presents a new diagnostic approach that could influence future therapeutic decision-making for the management of patients with PA.

Some researchers have emphasized that the control of biochemical levels as essential as blood pressure control in order to achieve good cardiovascular outcomes, whether amongst cohorts treated by adrenalectomy or medication (38–40). Hence, we used histology and clinical follow-up as the reference criteria in our current research to evaluate the accuracy of both PET/CT and AVS more objectively. In particular, we included cases with inconclusive subtyping diagnosis based on AVS results; these cases have been rarely discussed previously. ^68^Ga-Pentixafor PET/CT showed relatively high levels of sensitivity and specificity in the detection of functional lesions for most patients and played an important role in clinical treatment and prognostic prediction.

There are several limitations to our study that need to be considered. First, as a prospective study carried out in a single center, our study features inherent bias in terms of selection. For example, the proportion of unilateral lesions in our study was higher than that of bilateral lesions; one reason for this is that patients with a more severe phenotype were more likely to be referred to our centers. In addition, our sample size was small. Larger scale studies are now needed. Second, we cannot ascertain false negative test results since it would be unethical to perform operations on all patients. Third, the follow-up period was not long enough to compare the final probability of cardiovascular events in patients whose treatment was directed by ^68^Ga-Pentixafor PET/CT or AVS.

## Conclusion

Our prospective clinical trial found that ^68^Ga-Pentixafor PET/CT functional imaging represents a novel and reliable tool for PA subtype diagnosis for both functional lateralization and follow-up outcomes. This method provides a means of non-invasive diagnosis for most patients with PA and offers a universal diagnostic alternative to AVS, thus reducing several complicated invasive operations. Furthermore, ^68^Ga-Pentixafor PET/CT identified additional cases of unilateral surgically curable PA for which AVS failed to perform classification. Based on this, we recommend ^68^Ga-Pentixafor PET/CT as a first-line test for future classification. Our findings should be verified in a larger population.

## Data availability statement

The original contributions presented in the study are included in the article/**Supplementary Material**. Further inquiries can be directed to the corresponding authors.

## Ethics statement

The studies involving humans were approved by Ethics Committee of National Medical Research Center, Second Xiangya Hospital, Central South University. The studies were conducted in accordance with the local legislation and institutional requirements. The participants provided their written informed consent to participate in this study.

## Author contributions

XY: Data curation, Investigation, Writing – original draft, Writing – review & editing. KA: Data curation, Formal analysis, Writing – original draft. JL: Data curation, Formal analysis, Writing – original draft. WL: Data curation, Resources, Validation, Writing – review & editing. XM: Project administration, Supervision, Validation, Writing – review & editing. LZ: Investigation, Software, Visualization, Writing – review & editing. XX: Software, Visualization, Writing – review & editing. XS: Conceptualization, Project administration, Validation, Writing – review & editing. YW: Conceptualization, Funding acquisition, Project administration, Resources, Supervision, Writing – review & editing. YL: Conceptualization, Funding acquisition, Resources, Supervision, Writing – review & editing.
