# Supplementary material for: A comparison of the performance of 68Ga-Pentixafor PET/CT versus adrenal vein sampling for subtype diagnosis in primary aldosteronism
Source: Front Endocrinol (Lausanne). 2024 Feb 14;15:1291775. doi: 10.3389/fendo.2024.1291775 (PMC10899670; doi:10.3389/fendo.2024.1291775)
Supplement: Supplementary file 1 [file DataSheet_1.docx]

**Supplementary** **Materials**

**Primary hyperaldosteronism (PA) diagnosis process and criteria**

Before screening, all patients were evaluated after discontinuing all antihypertensive drugs, except for non-dihydropyridine calcium channel blockers and alpha-adrenergic blockers, such as spironolactone, eplerenone (mineralocorticoid receptor antagonist) and potassium (K) diuretics for at least 4 weeks, and beta blockers, angiotensin-converting enzyme inhibitors and angiotensin receptor antagonists for at least 2 weeks. Renin-angiotensin-aldosterone system (RAAS) screening included measurement of the plasma aldosterone concentration (PAC) and plasma renin activity (PRA) and calculation of the aldosterone-renin ratio (ARR). Patients with a PAC ≥ 15 ng/dl and an ARR ≥ 30 ng/dl/ng/ml/h underwent a further captopril challenge test (CCT) and/or saline infusion test (SIT). Diagnostic criteria were as follows: After CCT, patients who met all of the following criteria were diagnosed with PA: (1) PAC decreased < 30 %, (2) ARR maintained ≥ 30 ng/dl/ng/ml/h, and (3) PAC was ≥ 11 ng/dl. After SIT, patients who met post-infusion plasma aldosterone levels > 10 ng/ dl were diagnosed with PA.[1–3]

**Adrenal vein sampling**

AVS should be performed after at least 3 h in the recumbent position. We use bolus and all the patients performed both basal and stimulated procedure. All AVS procedures were performed by the same experienced interventional radiologist at Xiangya Second Hospital of Central South University. Samples were obtained simultaneously from the inferior vena cava (IVC) and both adrenal veins (AVs) before and 10 minutes after ACTH stimulation. ACTH (produced by Shanghai No. 1 Biochemical and Pharmaceutical Corp) was injected as a 0.125 mg (25 U) bolus. The catheter was inserted to the adrenal vein via a percutaneous femoral approach. The placement of the tip of the catheter was confirmed by injection of a small amount of contrast agent, and blood samples were obtained by gravity or slight negative pressure. The success of catheterization was determined by the selectivity index (SI; derived from AV/IVC cortisol concentrations) ≥ 2 before and ≥ 3 after ACTH stimulation. The source of excessive aldosterone was assessed by the lateralization index (LI) which was calculated by dividing the dominant adrenal cortisol corrected aldosterone level (A/C)DOM by that of the opposite side (A/C)OPP. UPA was diagnosed if LI was ≥ 2 before and ≥ 4 after ACTH administration.[4,5]

**68Ga-Pentixafor Synthesis**

The precursor (obtained from CSBio Co., CA, USA) was dissolved in ultrapure water to 1 mg/ml before use. 68GaCl3 was eluted from an 68Ge/68Ga generator using 0.05 M HCl and adjusted pH to 4.5 with 1.00 M sodium acetate (NaAc) buffer. The mixture was then transferred to a sterilized vial containing 20 μg of Pentixafor and heated at 95 °C for 10 min. The reaction mixture was diluted with 20 ml sterile water, and passed through a Sep-Pak C18 Light cartridge (Waters, USA). The product was eluted from the cartridge with 1 mL 65% ethanol and diluted with 9 ml saline. The solution was then injected through a 0.22 μm aseptic filtration membrane and collected to a sterilized vial for later injection. Radioactive HPLC was used to assess the radiochemical purity. The radiochemical purity of the 68Ga-Pentixafor product exceeded 99.5%.

**Immunohistochemistry**

Following semi-quantitative analysis, the assigned staining intensity scores were 0, 1, 2 and 3, corresponding to negative, weak, moderate, and strong staining intensity, respectively. The score corresponding to the proportion (%) of positive cells was as follows: score 0, 0%; score 1, 1%–9%; score 2, 10%–49%; score 3, 50%–74%; score 4, 75%–100%. The value of the semi-quantitative h score was calculated by multiplying the intensity score by the percentage score. The h score was divided into three levels: low (0–4), medium (4–8) and high (8–12).

**Supplementary Figure 1.** The performances of 68Ga-Pentixafor uptakes based on adrenal glands.


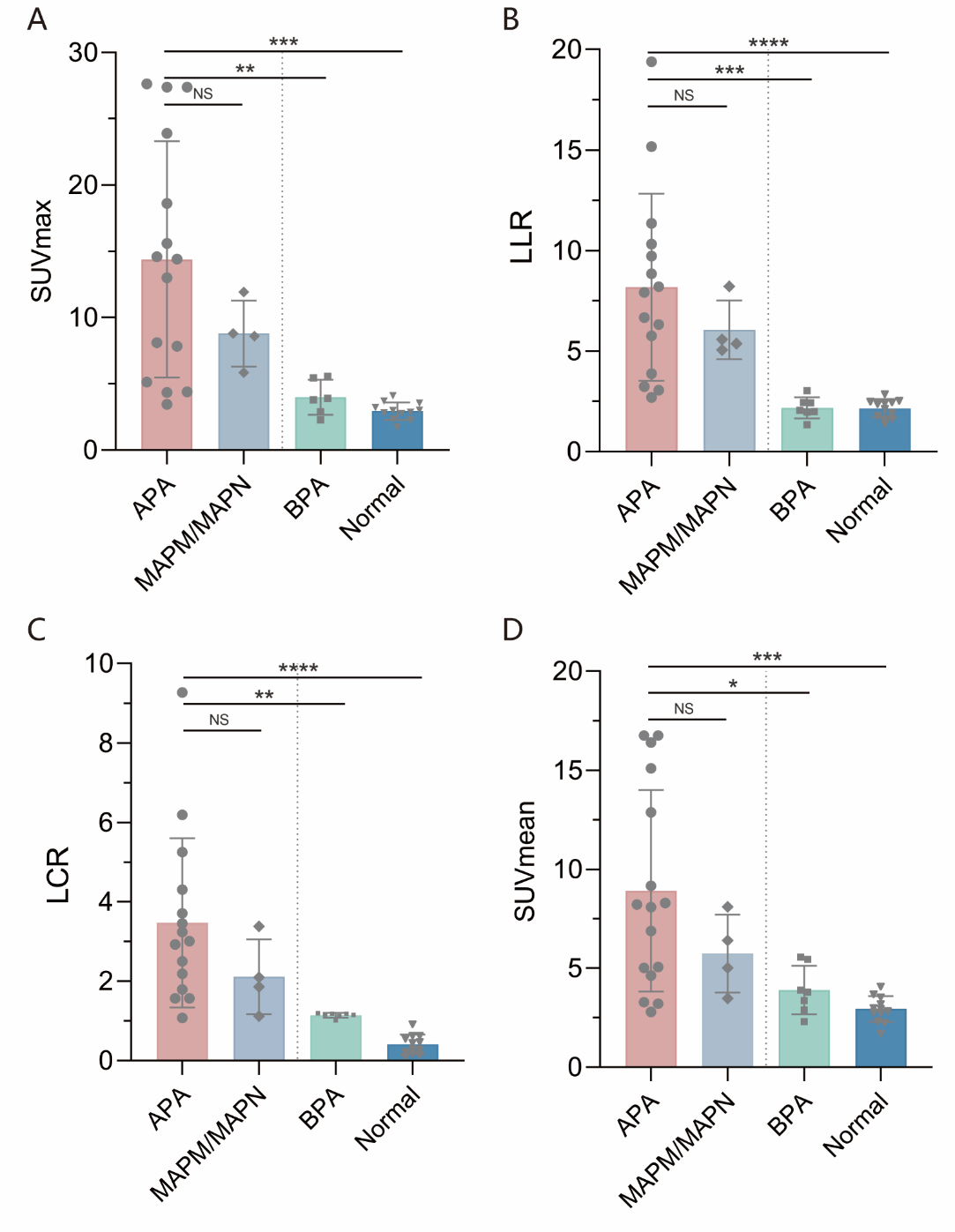


(A) SUVmax for 68Ga-Pentixafor uptake based on the adrenal glands. APA lesions had a significantly higher SUVmax than MAPM/MAPN and BPA (14.4 vs. 8.8 and 4.0, P < 0.05, respectively). The uptake of normal adrenals had the lowest SUVmax of 2.9. The SUVmax of lesions in the surgery group (left of the dotted line) was considerably higher in comparison with lesions in the medication group (13.2 vs 3.9, P < 0.01). (B, C and D) The performance of other uptake ratios for 68Ga-Pentixafor PET/CT. APA, aldosterone-producing adenoma; MAPM/MAPN, multiple aldosterone-producing nodules/micronodules; BPA, bilateral primary aldosteronism; SUVmax, maximum standardized uptake value. LCR, ratio of lesional SUVmax to contralateral adrenal SUVmean; LLR, ratio of lesional SUVmax to normal liver SUVmean.

**Supplementary Figure 2.** Correlation between uptake values for 68Ga-Pentixafor and partial clinical characteristics


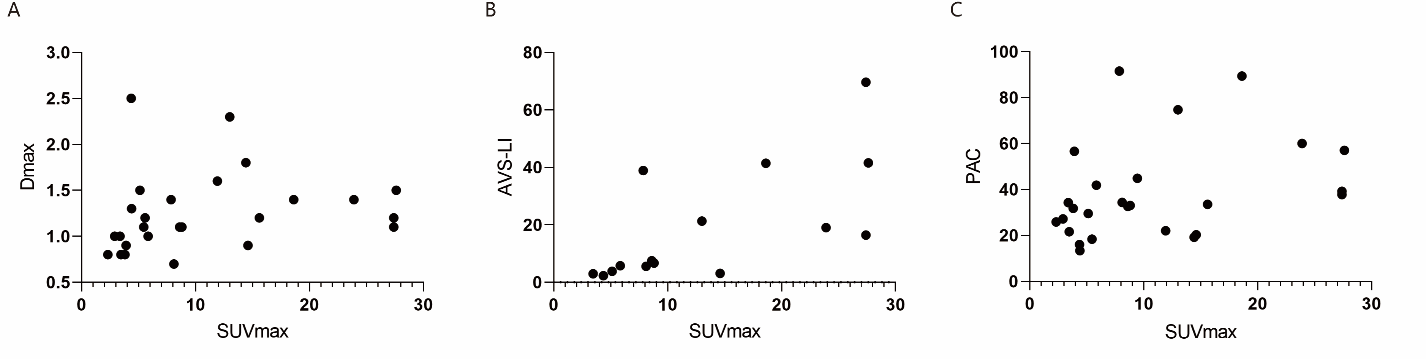


Dmax, maximum diameter; AVS-LI, lateralization index based on AVS; PAC, plasma aldosterone concentration.

**Supplementary Table 1.** Surgery outcomes assessment criteria for unilateral primary aldosteronism according to the Primary Aldosteronism Surgery Outcome (PASO) study[6]

|  | Complete success | Partial success | Absent success |
| --- | --- | --- | --- |
| Biochemical Outcome | Correction of hypokalaemia (if present pre-surgery) and normalisation of the aldosterone-to-renin ratio; in patients with a raised aldosterone-to-renin ratio post surgery, aldosterone secretion should be suppressed in a confirmatory test | Correction of hypokalaemia (if present pre-surgery) and a raised aldosterone-to-renin ratio: ≥ 50% decrease in baseline plasma aldosterone concentration (compared with pre-surgery) or abnormal but improved post-surgery confirmatory test result | Persistent hypokalaemia (if present pre-surgery) and/or persistent raised aldosterone-to-renin ratio with failure to suppress aldosterone secretion with a post-surgery confirmatory test |
| Clinical Outcome | Normal blood pressure without the aid of antihypertensive medication | Unchanged blood pressure surgery with less antihypertensive medication or a reduction in blood pressure with the same or less antihypertensive medication | Unchanged or increased blood pressure with the same or an increase in antihypertensive medication |

The time of follow-up was ≥ 6 months.

**Supplementary Table 2.** Individual data of all 26 patients recruited into study

| Patient  No. | Sex | Age | Lateralization side(s) | | | SUVmax | LCR | LLR | Dmax | Surgery | IHC | Biochemical  success | Clinical success |
| --- | --- | --- | --- | --- | --- | --- | --- | --- | --- | --- | --- | --- | --- |
|  |  |  | CT | AVS(LI) | PET |  |  |  |  |  |  |  |  |
| 1 | M | 57 | R | R(38.87) | R | 7.85 | 3.46 | 5.77 | 1.40 | R | Adenoma | Complete | Complete |
| 2 | M | 51 | L | L(5.76) | L | 5.84 | 3.38 | 8.23 | 1.00 | L | Hyperplasia | Complete | Complete |
| 3 | F | 60 | R | B(1.07L,0.93R) | R | 14.42 | 6.19 | 8.85 | 1.80 | R | Adenoma | Complete | Complete |
| 4 | M | 35 | B | R(41.40) | R | 18.63 | 5.25 | 11.36 | 1.40 | R | Adenoma | Complete | Complete |
| 5 | F | 48 | L | B(1.14L,0.91R) | B | 3.38 | 1.02 | 1.97 | 1.00 | - | - | - | - |
| 6 | F | 51 | L | L(41.58) | L | 27.63 | 9.27 | 15.18 | 1.50 | L | Adenoma | Complete | Complete |
| 7 | M | 26 | B | R(69.60) | R | 27.40 | 2.93 | 7.92 | 1.20 | R | Adenoma | Complete | Complete |
| 8 | F | 62 | B | B(0.61L,1.60R) | R | 11.93 | 1.86 | 5.06 | 1.60 | R | Hyperplasia | Complete | Partial |
| 9 | F | 70 | B | R(2.31) | R | 4.36 | 1.57 | 3.07 | 2.50 | R | Adenoma | Complete | Partial |
| 10 | M | 45 | L | R(2.96) | B | 3.46 | 1.08 | 2.70 | 0.80 | R | Adenoma | Complete | Complete |
| 11 | M | 54 | B | R(6.73) | B | 8.81 | 1.12 | 5.58 | 1.10 | R | Hyperplasia | Partial | Partial |
| 12 | M | 40 | L | B(1.84L,0.51R) | B | 5.57 | 1.17 | 2.46 | 1.20 | - | - | - | - |
| 13 | M | 54 | B | L(3.80) | L | 5.13 | 1.79 | 3.25 | 1.50 | L | Adenoma | Complete | Partial |
| 14 | F | 48 | L | L(21.27) | L | 13.01 | 4.31 | 10.33 | 2.30 | L | Adenoma | Complete | Complete |
| 15 | M | 63 | B | B(0.92L,1.11R) | B | 5.45 | 1.18 | 3.03 | 1.10 | - | - | - | - |
| 16 | F | 52 | L | L(5.54) | L | 8.10 | 2.19 | 6.33 | 0.70 | L | Adenoma | Complete | Complete |
| 17 | F | 56 | L | B(1.57L,0.64R) | L | 4.39 | 1.57 | 3.88 | 1.30 | L | Adenoma | Complete | Complete |
| 18 | M | 53 | R | R(7.47) | R | 8.60 | 2.10 | 5.38 | 1.10 | R | Hyperplasia | Complete | Complete |
| 19 | M | 56 | L | B(1.21L,0.82R) | B | 2.30 | 1.15 | 1.35 | 0.80 | - | - | - | - |
| 20 | M | 40 | B | R(19.05) | R | 23.90 | 2.50 | 19.40 | 1.40 | R | Adenoma | Complete | Complete |
| 21 | M | 49 | B | B(1.24L,0.82R) | B | 3.80 | 1.20 | 2.00 | 0.80 | - | - | - | - |
| 22 | M | 35 | B | B(1.13L,1.76R) | R | 15.60 | 3.71 | 8.21 | 1.20 | R | Adenoma | Complete | Complete |
| 23 | M | 35 | R | R(3.10) | R | 14.60 | 3.24 | 9.73 | 0.90 | R | Adenoma | Complete | Complete |
| 24 | M | 48 | L | B(1.56L,0.93R) | B | 3.90 | 1.18 | 2.44 | 0.90 | - | - | - | - |
| 25 | M | 60 | L | B(1.06L,0.94R) | B | 2.90 | 1.12 | 2.07 | 1.00 | - | - | - | - |
| 26 | M | 59 | R | R(16.42) | R | 27.40 | 3.01 | 6.68 | 1.10 | R | Adenoma | Complete | Complete |
|  |  |  |  |  |  |  |  |  |  |  |  |  |  |

SUVmax, maximum standardized uptake value; LCR, ratio of lesional SUVmax to contralateral adrenal SUVmean; LLR, ratio of lesional SUVmax to normal liver SUVmean; Dmax, maximum diameter; M, male; F, female; R, right; L, left; B, bilateral; C, complete success; P, partial succes

**Supplementary Table 3.** Characteristics of six patients with discordant lateralization in PET/CT and AVS

| Patient No. | #3 | #8 | #10 | #11 | #17 | #22 |
| --- | --- | --- | --- | --- | --- | --- |
| BMI (kg/m^2^) | 23.1 | 28.5 | 28.5 | 30.8 | 23.5 | 28.5 |
| CT findings | Right | Bilateral | Left | Bilateral | Left | Bilateral |
| Left | Normal | Multiple nodules | Hyperplasia | Multiple nodules | Multiple nodules | Hyperplasia |
| Right | Single nodule | Single nodule | Normal | Multiple nodules | Normal | Single nodule |
| AVS (LI) | B(1.07L, 0.93R) | B(0.61L, 1.60R) | R(2.96) | R(6.73) | B(1.57L, 0.64R) | B(1.13L, 1.76R) |
| PET lateralization | Right | Right | Bilateral | Bilateral | Left | Right |
| SUVmax | 14.42 | 11.93 | 3.46 | 8.81 | 4.39 | 15.6 |
| LCR | 6.19 | 1.86 | 1.08 | 1.12 | 1.57 | 3.71 |
| Surgery side | Right | Right | Right | Right | Left | Right |
| IHC by HISTALDO | APA | MAPN | APA | MAPN | APA | APA |
| Baseline |  |  |  |  |  |  |
| Systolic BP (mmHg) | 180 | 165 | 220 | 230 | 165 | 180 |
| Diastolic BP (mmHg) | 120 | 106 | 132 | 100 | 90 | 121 |
| Serum potassium (mmol/l) | 3.61 | 3.36 | 2.82 | 3.16 | 2.89 | 1.99 |
| PAC (ng/dl) | 19.2 | 22 | 21.6 | 33 | 13.4 | 33.5 |
| PRA (ng/ml/h) | 0.19 | 0.12 | 0.07 | 0.08 | 0.13 | 0.33 |
| ARR ([ng/dl]/[ng/ml/h]) | 101.05 | 183.33 | 308.57 | 412.50 | 103.08 | 101.52 |
| Follow-up |  |  |  |  |  |  |
| Systolic BP (mmHg) | 136 | 132 | 136 | 160 | 122 | 125 |
| Diastolic BP (mmHg) | 87 | 80 | 89 | 92 | 80 | 79 |
| Serum potassium (mmol/l) | 4.77 | 4.58 | 4.2 | 3.52 | 3.66 | 4.11 |
| PAC (ng/dl) | 5 | 8.2 | 33.8 | 3.9 | 6.7 | 3.7 |
| PRA (ng/ml/h) | 2.58 | 1.80 | 2.55 | 0.16 | 0.38 | 0.52 |
| ARR ([ng/dl]/[ng/ml/h]) | 1.94 | 4.56 | 13.25 | 24.38 | 17.63 | 7.12 |
| Remarks | With concurrent hypercortisolism | - | - | - | - | - |

Nodule: if adrenal lesion is round or oval, smoothed, well defined, and ≥4 mm in diameter; Hyperplasia: if adrenal gland thickness measured ≥10 mm in diameter.[7,8]

**Supplementary Table 4.** The relationships of other 68Ga-Pentixafor uptake values with clinical features

| Clinical features | Correlation coefficients | | |
| --- | --- | --- | --- |
|  | LCR | LLR | SUVmean |
| Age | -0.18 | -0.31 | -0.37 |
| BMI | -0.51** | -0.38 | -0.08 |
| Systolic pressure | -0.24 | 0.01 | -0.01 |
| Diastolic pressure | 0.11 | 0.25 | 0.31 |
| Dmax | 0.51** | 0.46* | 0.45* |
| AVS-LI# | 0.68** | 0.63* | 0.81 |
| Serum potassium | -0.17 | -0.05 | -0.14 |
| PAC | 0.40* | 0.42* | 0.50** |
| ARR | 0.006 | -0.005 | 0.02 |

**P <0.01; *P <0.05

#: among patients who were diagnosed with UPA. BMI, body mass index; ARR, aldosterone-renin ratio.

**Reference**

1. Lee FT, Elaraj D. Evaluation and Management of Primary Hyperaldosteronism. Surg Clin North Am. 2019;99:731–45.

2. Byrd JB, Turcu AF, Auchus RJ. Primary Aldosteronism: Practical Approach to Diagnosis and Management. Circulation. 2018;138:823–35.

3. Reincke M, Bancos I, Mulatero P, Scholl UI, Stowasser M, Williams TA. Diagnosis and treatment of primary aldosteronism. The Lancet Diabetes & Endocrinology. 2021;9:876–92.

4. Hongyun L, Ping L, Shanmei S, Xuebin Z, Wenhuan F, Hong H, et al. Role of adrenal vein sampling in differential diagnosis of primary aldosteronism subtypes. National Medical Journal of China. 2017;97:3291–6.

5. Dekkers T, Prejbisz A, Kool LJS, Groenewoud HJMM, Velema M, Spiering W, et al. Adrenal vein sampling versus CT scan to determine treatment in primary aldosteronism: an outcome-based randomised diagnostic trial. Lancet Diabetes Endocrinol. 2016;4:739–46.

6. Williams TA, Lenders JWM, Mulatero P, Burrello J, Rottenkolber M, Adolf C, et al. Outcomes after adrenalectomy for unilateral primary aldosteronism: an international consensus on outcome measures and analysis of remission rates in an international cohort. The Lancet Diabetes & Endocrinology. 2017;5:689–99.

7. Influence of Adrenal Venous Sampling on Management in Patients with Primary Aldosteronism Independent of Lateralization on Cross-Sectional Imaging - PubMed [Internet]. [cited 2023 Nov 2]. Available from: https://pubmed.ncbi.nlm.nih.gov/30930101/

8. Vincent JM, Morrison ID, Armstrong P, Reznek RH. The size of normal adrenal glands on computed tomography. Clin Radiol. 1994;49:453–5.
